# Neuron arbor geometry is sensitive to the limited-range fractal properties of their dendrites

**DOI:** 10.3389/fnetp.2023.1072815

**Published:** 2023-01-25

**Authors:** Conor Rowland, Julian H. Smith, Saba Moslehi, Bruce Harland, John Dalrymple-Alford, Richard P. Taylor

**Affiliations:** ^1^ Physics Department, University of Oregon, Eugene, OR, United States; ^2^ School of Pharmacy, University of Auckland, Auckland, New Zealand; ^3^ School of Psychology, Speech and Hearing, University of Canterbury, Christchurch, New Zealand; ^4^ New Zealand Brain Research Institute, Christchurch, New Zealand

**Keywords:** neurons, fractal analysis, fractal dimension (D), tortuosity, connectivity, neuromorphology, confocal microscopy, hippocampal CA1

## Abstract

Fractal geometry is a well-known model for capturing the multi-scaled complexity of many natural objects. By analyzing three-dimensional images of pyramidal neurons in the rat hippocampus CA1 region, we examine how the individual dendrites within the neuron arbor relate to the fractal properties of the arbor as a whole. We find that the dendrites reveal unexpectedly mild fractal characteristics quantified by a low fractal dimension. This is confirmed by comparing two fractal methods—a traditional “coastline” method and a novel method that examines the dendrites’ tortuosity across multiple scales. This comparison also allows the dendrites’ fractal geometry to be related to more traditional measures of their complexity. In contrast, the arbor’s fractal characteristics are quantified by a much higher fractal dimension. Employing distorted neuron models that modify the dendritic patterns, deviations from natural dendrite behavior are found to induce large systematic changes in the arbor’s structure and its connectivity within a neural network. We discuss how this sensitivity to dendrite fractality impacts neuron functionality in terms of balancing neuron connectivity with its operating costs. We also consider implications for applications focusing on deviations from natural behavior, including pathological conditions and investigations of neuron interactions with artificial surfaces in human implants.

## 1 Introduction

The term “fractal” was introduced in 1975 to highlight similarities between a diverse range of natural objects and the scale invariant properties of mathematical patterns researched over the previous century (Mandelbrot and Pignoni, 1983). Fractal dimension, *D*, has since emerged as a powerful tool for quantifying the fractal repetition of patterns at multiple size scales and how this scale invariance impacts the visual and functional properties of many natural objects (Bassingthwaighte et al., 1994; Iannaccone and Khokha, 1996). Fractal branches are particularly prevalent in nature. In addition to trees populating many natural environments, animals benefit from these structures in, for example, their bronchial trees (Nelson et al., 1990; Lennon et al., 2015) and neural networks (Di Ieva, 2016; Smith et al., 2021). As their branches spread out in space, the structure of the resulting arbor features two embedded fractal patterns– the branches and the gaps forming between them. Their structural relationship offers the potential to balance functionality with operational costs whilst also maintaining structural integrity. In this balancing process, the repeating patterns offer large interfaces to interact with light in the case of trees (Seidel et al., 2019) and oxygen in the case of bronchial trees (Hou et al., 2010). Neurons present an extra level of intricacy because they connect to fractal neighbors to transmit signals through a network.

Exact fractals, assembled mathematically by repeating patterns precisely at many scales, serve as a useful model for picturing how branches influence the distribution of gaps. Branches with lower *D* values reduce their branch lengths at much faster rates between repeating iterations, such that their arbors are spacious when compared to the dense structures generated by higher *D* values (Supplementary Figure S1). The dendritic branches of neurons are considerably more subtle, however. Randomness disrupts the exact repetition. Consequently, only their patterns’ statistical qualities repeat. Furthermore, for the neurons examined in our study the scaling range for this repetition is limited by the arbor (at the coarse scale) and branch (at the fine scale) widths.

Given these constraints, we recently investigated the degree to which neurons are fractal and the origin of this fractality. By analyzing three-dimensional images of pyramidal neurons in the CA1 region of the rat hippocampus, we showed that, despite being named after trees, the dendrites of a neuron are considerably different in their scaling behavior (Smith et al., 2021). Whereas trees are traditionally modeled using a fractal distribution of branch lengths (Mandelbrot and Pignoni, 1983; Oppenheimer, 1986; Frame and Urry, 2016), the ways in which the dendrites fork and weave through space are important for determining the scale-invariant character of the arbor’s branches and gaps. Previous studies of neuron connectivity and dendritic cost considered component parameters of the neuron geometry such as the dendrites’ weave quantified using tortuosity, branch length, and an analysis of self-similar scaling of small parts of the arbor (Wen et al., 2009). In contrast, we showed that the arbor’s fractal dimension, *D*_*A*_, incorporates these parameters in an integrative approach that directly reflects the fractal-like geometry across multiple dendrites and multiple size-scales of the neuron’s entire arbor. By measuring the relative contributions of coarse and fine scale patterns within the arbor, *D*_*A*_ successfully mapped the optimization of neuron functionality even though the scaling behavior lacked the infinite range associated with mathematical fractals. The competing constraints examined within this optimization process were the dendrites’ potential to connect to other neurons (characterized by their physical profiles) along with the costs associated with building (mass) and operating (metabolic energy) the dendrites.

Given the geometric complexity originating from the interplay between the dendrite length distributions and their forking and weave angles, in the current work we seek to clarify the origin of the neuron’s fractal behavior by examining how the individual dendrites relate to the fractal properties of the whole arbor. The dendritic arbor of our neurons features two component arbors (apical and basal) and in this study we focus on the basal arbor, which is composed of 32 dendrites on average. Our comparison of dendrites across 105 arbors shows that, irrespective of their lengths, the individual dendrites typically reveal very mild fractal characteristics quantified by low fractal dimensions, *D*_*B*_, close to those expected for straight lines. This behavior is so mild that we sought confirmation by comparing two fractal methods—a traditional method employed in the first demonstration of nature’s fractality (Richardson, 1961), and a novel method that uses tortuosity, *T* (Wen and Chklovskii, 2008; Ledderose et al., 2014), to quantify the meandering nature of the dendrites across multiple scales. The high degree of agreement between the two methods in their measurement of *D*_*B*_ emphasizes the appropriateness of characterizing the scaling properties of dendrites using fractal geometry. In addition to providing confirmation of *D*_*B*_, this second method also allows an examination of the relationship between *D*_*B*_ and the scaling behavior of *T*. By doing so, we unite traditional and novel approaches to understanding neuron geometry. In particular, our results facilitate connections between the fractal community (familiar with *D*) and the neuroscience community (familiar with *T*).

By using models that distort the way that the dendrites weave and fork, we show that deviations from their natural shapes induce distinct changes in the arbor’s fractal dimension, *D*_*A*_, through the interplay of the branches and their gaps. Furthermore, the size of these changes in the arbor depends on the character of the dendrite distortions. For example, for the deviations of the rat hippocampal neurons used in the current study, the sensitivity of *D*_*A*_ to changes in *D*_*B*_ depends on whether the dendrites are straightened to reduce *D*_*B*_ or made more meandering to increase *D*_*B*_. Given the dependence of neuron connectivity to *D*_*A*_ established previously (Smith et al., 2021), our current study highlights the degree to which neuron functionality relies critically on the fractal arrangement of the dendrites even for neuron types that are only mildly meandering. Significantly, this functionality exhibits a well-defined dependence on *D*_*B*_ even though the fractal scaling behavior of the dendrites occurs over a highly limited range of size scales. Although lacking the scaling range associated with mathematical fractal exponents, *D*_*B*_ therefore serves as an “effective” fractal dimension for quantifying the neurons’ physical form. Given this contrast between our physical neurons and infinitely repeating mathematic fractals, we will discuss our results within the context of previous debates over “limited-range” fractals. Our results are also of interest to fundamental neuroscience research relating form to function of healthy neural networks and builds on Ramón y Cajal’s wiring economy principle from a century ago (RamónCajal, 1999). In addition, the results are important to applications which quantify changes from natural behavior, for example in pathological changes and in neuron interactions with artificial surfaces.

## 2 Materials and methods

### 2.1 Rodents

The study was conducted in accordance with ARRIVE guidelines. Rat pups were bred and housed with their mother in cages with wood chips and *ad libitum* food and water in an environmentally controlled room. All procedures pertaining to the use of live rats were conducted in compliance with all relevant ethical regulations for animal testing and research and were approved by the University of Canterbury Animal Ethics Committee, 2008-05R.

### 2.2 Image acquisition, model reconstruction, and model distortion

**FIGURE 1:**
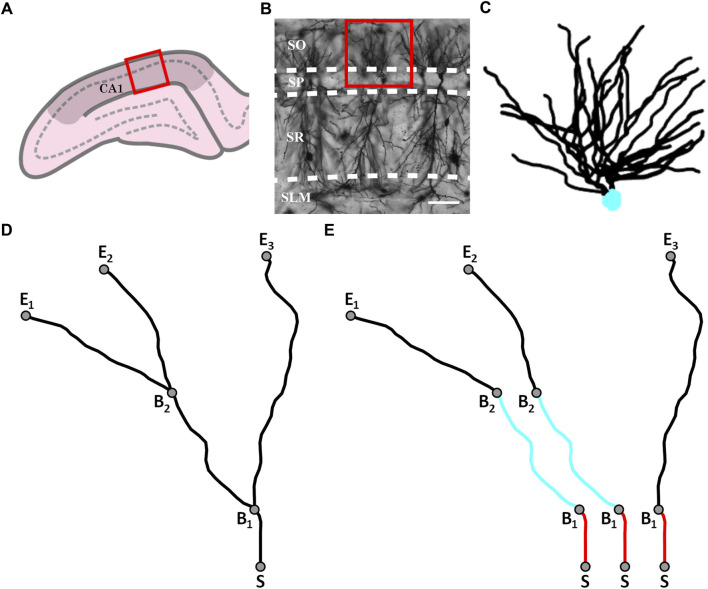
**(A)** Schematic diagram of a coronal slice through the hippocampus at Bregma −4.52 mm showing a selected location (red box) within the hippocampal CA1 region (darkened area); the pyramidale layer is denoted by the dashed line. **(B)** An example confocal micrograph taken from within the selected location highlighted in **(A)** spanning the oriens (SO), pyramidale (SP), radiatum (SR), and lacunosum-moleculare (SLM) strata. The red box in the micrograph highlights the approximate region occupied by the basal arbor of a single neuron and the white scale bar corresponds to 100 μm. **(C)** An example reconstruction of a neuron’s basal arbor with the neuron’s soma colored in cyan and its dendrites in black. **(D)** An example of the paths taken by a neuron’s dendrites. S denotes the point where the dendrites initially extend out of the soma. B_1_ and B_2_ denote the points at which the dendrites bifurcate. E_1_, E_2_, and E_3_ denote the endpoints of the dendrites. **(E)** The three dendritic branches seen in **(D)** separated from one another. The red and cyan colors are used to indicate sections that are shared between branches.

Thirty-three adult PVGc male hooded rats (13–16 months old) were given an overdose of sodium pentobarbital. The brains were removed fresh without perfusion, rinsed with Milli-Q water, and a 4 mm block containing the hippocampus was cut in the coronal plane using a brain matrix (Ted Pella, Kitchener, Canada). These tissue blocks were processed with a metallic Golgi-Cox stain, which stains 1%–5% of neurons so that their cell bodies and dendritic arbors can be visualized. 200 µm thick coronal brain sections spanning the bilateral dorsal hippocampus were taken using a microtome. A standard microscope was used to locate isolated neurons in the dorsal CA1 subfield (Figure 1A). A Leica laser scanning confocal microscope was used to collect high-resolution image stacks for these arbors. An example of one of the images comprising a stack is shown in Figure 1B. The image stacks were captured using a ×20 glycerol objective lens with a 0.7 numerical aperture, providing an x and y resolution of 0.4 µm. The step size (z distance between image stacks) was 2 µm.

Arbors were manually traced through the image stacks using Neurolucida (MBF Bioscience, Williston, VT, United States) (Neurolucida, 2019) to create three-dimensional models (Figure 1C). The models were then exported to the Wavefront (.obj) format and their soma removed, leaving only the arbor’s dendrites. In this format, the arbor reconstructions were defined by sets of connected, cylindrical segments. The median length and width of the segments were 2.4 μm and 1.4 µm, respectively. The weave angles, *θ*, were defined as the angles between connecting segments along a branch and the forking angles, *ϕ*, as the first weave angle following a bifurcation point on a branch. We then created distorted versions of the neuron models by mathematically manipulating the branch weave and forking angles through a process that multiplied every *θ* and *ϕ* value by a common factor, *α*. The range of *α* values used (0.5–2 in steps of 0.25) was chosen to ensure that separate branches rarely intersected, so ensuring a physically reasonable condition. Further details of this distortion technique have been reported elsewhere (Smith et al., 2021). The analysis of all models was performed by authors who were blinded to rat ID numbers.

### 2.3 Calculating the coastline fractal dimension of a branch

We define a dendritic branch as any path that starts from the soma and ends at the tip of a dendrite (Figure 1D). Within this definition, we emphasize that different branches commonly have shared sections (Figure 1E). We can then specify the various parameters summarized in Table 1 that are related to the branch’s geometry.

**TABLE 1:** List of geometric parameters used throughout this article.

Parameter	Definition
*θ*	Branch weave angle
*ϕ*	Branch forking angle
*α*	Angle multiplier
*R*_*A*_	Arbor radius
*L*_*R*_	Ruler length
*L*_*F*_	Ruler length set to the finest resolution
*L*_*E*_	Ruler length between the two ends of a branch
*L*_*D*_	Ruler length between the two ends of a chosen branch section
*L*_*T*_	Sum of the ruler lengths spanning a chosen branch section
*L*_*P*_	Path length (curvilinear length of a path along a chosen branch section)
*L*_*B*_	Maximum *L*_*P*_ (the path length along an entire branch)
*L*_*box*_	Box length used in the box-counting analysis
*L*_*grid*_	Largest of the three side lengths for the smallest grid enclosing a neuron’s arbor
*T*	Tortuosity (*L*_*P*_/*L*_*D*_ measured between the two ends of a chosen branch section)
*D*_*A*_	Arbor fractal dimension measured using the box-counting analysis
*D*_*BC*_	Branch fractal dimension measured using the coastline analysis
*D*_*BCN*_	Branch fractal dimension measured using the normalized coastline analysis
*D*_*BT*_	Branch fractal dimension measured using the tortuosity analysis

**FIGURE 2:**
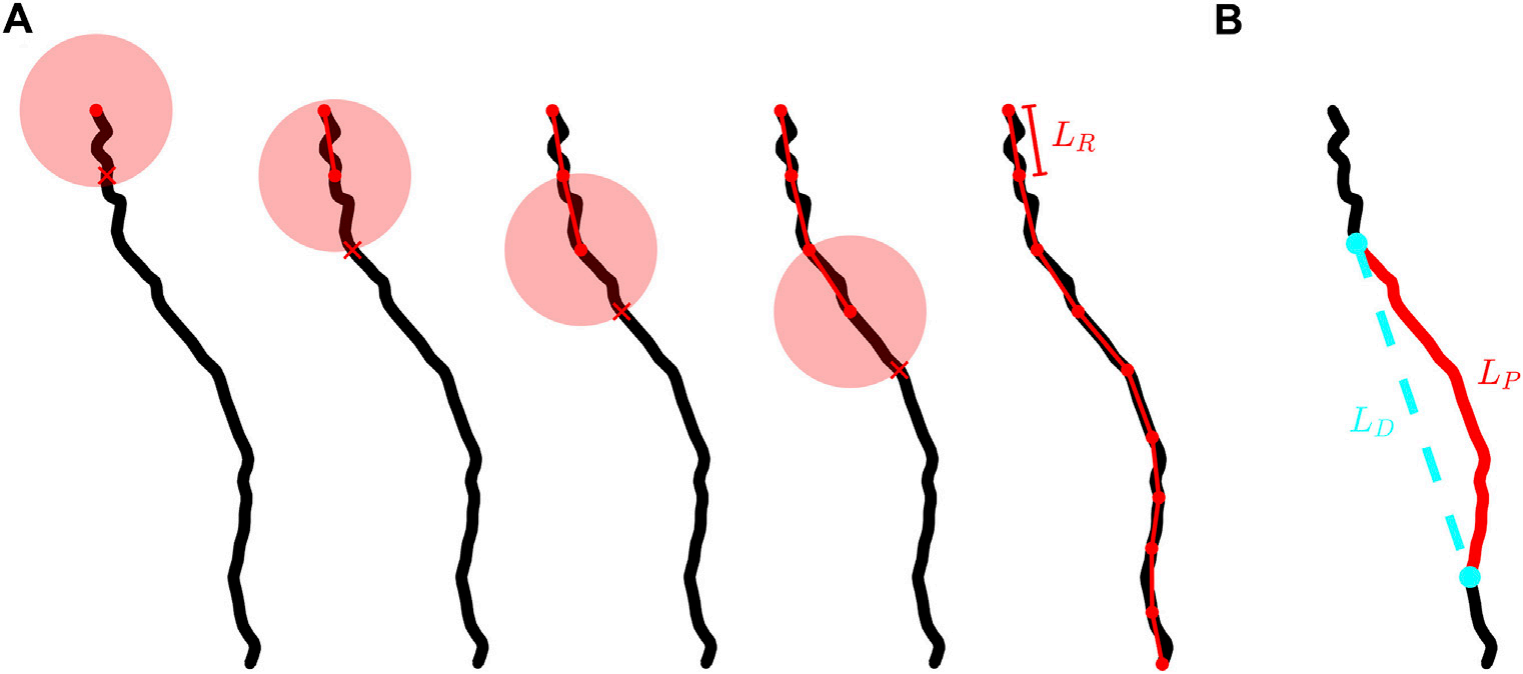
**(A)** Schematic demonstrating the placement of rulers along the branch of a neuron. The black curve shows an example neuron branch, the connected red dots show the segmented version of a branch at a given ruler length, *L*_*R*_, the transparent red circles represent the spherical shells used to determine where to place each segment, and each red X indicates where the branch intersects the spherical shell. We note that the radius of each spherical shell is equal to *L*_*R*_. **(B)** The same branch shown in **(A)** with a chosen branch section of path length, *L*_*P*_, highlighted in red and the Euclidean distance, *L*_*D*_, separating the ends of this section shown by the dashed cyan line.

We employ a three-dimensional extension of the traditional method pioneered by Richardson (Richardson, 1961) and then Mandelbrot (Mandelbrot, 1967) in their discovery of the fractal character of meandering coastlines. The “coastline method” examines the branch at different resolutions through its employment of a ruler of length *L*_*R*_. Shown in Figure 2A, the branch is segmented into a series of rulers. The branch’s fractal scale invariance can then be revealed through the power law dependence of the number of rulers needed to span the branch’s entire length, *N*, on *L*_*R*_. The exponent of $N\propto {L_{R}}^{-D_{BC}}$ is labelled as the branch’s coastline fractal dimension, *D*_*BC*_, and can be extracted from a log-log plot of *N* versus *L*_*R*_. In our analysis, we normalize *L*_*R*_ using *L*_*E*_ (the largest possible ruler length connecting the soma to the branch endpoint).

This fractal scaling is limited at fine scales by the finite sizes of the branches which set a fine scale “cut-off”. We do not consider rulers smaller than 4 µm (which approaches the median value of the branch segment length) because smaller rulers would start to detect the linear character of the cylindrical segments rather than the fractal character of the meandering branches. At the course scale, we allow for rulers that span up to 40 μm, which provides an order of magnitude scaling on the log-log plots used to extract *D*_*BC*_. We apply this “one-order” rule to all branch sizes to standardize the fitting procedure that generates their *D*_*BC*_ values. One order of magnitude is chosen to maximize the number of branches used in the comparison of our two methods for measuring branch fractal dimension: this range excludes only 35 of the 3,354 total undistorted branches, compared to, for example, 1930 branches if 1.5 orders is used as the scaling range.

Figure 2A provides a graphical representation of how the segmented versions of a branch are generated. We start by centering a spherical shell of radius *L*_*R*_ on the branch end connected to the soma. The start point of the first segment is set at this location and the end point is set where the branch intersects the spherical shell. If the spherical shell intersects the branch at multiple locations, then the location which has the shortest path length along the branch from the intersection to the center of the spherical shell is chosen. The next segment is defined using the same process but the spherical shell is instead centered at the end point of the previous segment. However, once the first segment is placed, parts of the branch that have already been segmented but which intersect the shell are not considered when placing a new segment. This ensures that in general each new segment is placed closer to the branch terminal point than the previous segment. This process is repeated until the spherical shell no longer intersects the branch. If part of the branch remains unaccounted for then a truncated segment is inserted to connect the endpoint of the previous segment to the terminal point. For cases when a truncated segment exists at the terminal point, the truncated segment is counted as a fraction of a segment. For example, for a ruler length of 10 μm, if the segmented version of the branch is comprised of 12 full segments and a truncated segment of length 2 µm then *N* is counted as 12.2.

The above analysis is performed on both the undistorted and distorted neurons. For the undistorted neurons, we also employ a method for calculating the normalized coastline fractal dimension, *D*_*BCN*_, of all the branches within a given neuron. Normalizing *L*_*R*_ to *L*_*E*_ allows for a direct comparison of *N* across branches with different lengths. We calculate *D*_*BCN*_ by plotting all of the branches on a single log-log graph of *N* against *L*_*R*_/*L*_*E*_ and extracting the magnitude of the slope of the combined data. In order to avoid having some branches dominate the fine and coarse scales of the fit, only the range of *L*_*R*_/*L*_*E*_ over which all of the neuron’s branches contribute is used in the fitting procedure (which spans 0.75 orders of magnitude). We stress that this normalization procedure is used only to demonstrate the similar fractal character of different-sized branches. Subsequent analysis focuses on *D*_*BC*_ rather than *D*_*BCN*_ values.

### 2.4 Calculating the tortuosity and tortuosity fractal dimension of a branch

The definition of tortuosity, *T*, that we adopt in this paper is the ratio of a path’s curvilinear length, *L*_*P*_, to the Euclidean distance between the two endpoints of that path, *L*_*D*_. We define a path as any section of a neuron’s branch connecting two points on that branch. An example path and its associated *L*_*P*_ and *L*_*D*_ lengths are shown in Figure 2B. *L*_*P*_ is calculated by summing the lengths of all of the cylindrical segments spanning the chosen section of the branch. In terms of ruler measurement, *L*_*P*_ approximates to the total length of all rulers spanning the chosen path with the ruler set to the smallest resolution. *L*_*P*_ can in principle be used to measure the length of any branch section - from the smallest sections approaching the segment length through to the largest possible section when *L*_*P*_ = *L*_*B*_, where *L_B_* is the path length of an entire branch. In each case, *L*_*P*_ captures the fractal tortuosity (i.e. meandering) of the branch. In contrast, *L*_*D*_ represents the length of the straight, Euclidean line connecting the two ends of the chosen section along the branch. We measure *T* across all possible paths along a branch and plot these versus *L*_*P*_ on a log-log graph. We then extract the tortuosity fractal dimension, *D*_*BT*_, of that branch using the relationships $T\propto {L_{P}}^{S}$ ; $D_{BT}=1/\left(1-S\right)$ (see Section 1 of the Supplementary Material).

Due to the large noise inherent in plots of *T* against *L*_*P*_ (as highlighted in Supplementary Figure S2), before fitting the data to extract *D*_*BT*_ we first divide it into bins over the desired scaling range (4 μm–40 µm is chosen to match the range examined in the coastline fractal analysis). We then calculate the average *T* value for each bin and fit the binned data. The slope of the resulting fit yields *D*_*BT*_ (using the above relationship to *S*) and for the chosen branch section. We also calculate a single *D*_*BT*_ value for all the branches across all neurons. This procedure is the same as that for calculating *D*_*BT*_ for an individual branch except that we combine the data from all the paths within all the branches across all neurons onto a single plot of *T* against *L*_*P*_. We note that duplicate paths exist due to different branches having overlapping sections (Figure 1E) and therefore remove any duplicated data before performing the fit.

### 2.5 Calculating the profile area, surface area, bounding area, and bounding volume of a neuron’s arbor

In order to quantify the potential for a neuron’s dendrites to connect to other neurons as well as the costs associated with building and operating those dendrites, we utilize the following metrics: profile area (*P*), surface area (*A*_*s*_), bounding area (*A*_*b*_), and bounding volume (*V*_*b*_). Each of these metrics are calculated for a given neuron’s arbor using MATLAB code developed in our previous study (Smith et al., 2021). Briefly, to measure *P* we orthogonally project a neuron’s arbor onto a 2-dimensional plane from a given viewing angle, uniformly expand the profile of this projection by 2 µm (to account for the potential growth of spines in the space around each dendrite), calculate the area of this expanded profile, and then average this expanded profile area over all possible viewing angles of the arbor. To measure *A*_*s*_, we calculate the surface area of an arbor by summing the area of all the triangular faces defining the cylindrical segments of the arbor’s dendrites. However, due to some faces being partially positioned inside the branches of an arbor, we employ a technique that measures *A*_*s*_ more precisely by increasing the resolution of the triangular faces and then removing those faces with all three corners inside a branch. Lastly, to measure *A*_*b*_ and *V*_*b*_, we calculate the surface area and volume, respectively, of the arbor’s convex hull.

## 3 Results

Figure 1B shows a representative image obtained using confocal microscopy of CA1 pyramidal neurons in the coronal plane of the dorsal rat hippocampus. Axonal and dendritic arbors extend from neuron somas located in the stratum pyramidale (SP) of the CA1 region. The basal arbor’s complex branching patterns extend into the neighboring stratum oriens (SO) where they collect signals from the axons of other neurons. Arbor sizes can be quantified using their radii (Caserta et al., 1995; Wen et al., 2009), and for our basal arbors the median arbor radius, *R*_*A*_, is ∼100 µm. Figure 1C shows an example three-dimensional reconstruction of an arbor. In principle, each dendrite could extend into the SO layer following a perfectly straight line with dimension *D* = 1 or meander along a very winding trajectory that completely fills space with a dimension of *D* = 3. If the arbor features fractal dendrites instead of these integer dimensions characterizing Euclidean shapes, then each of these will be quantified by an intermediate *D* value lying between 1 and 3. Fractals with larger contributions of fine patterns will have higher *D*_*B*_ values than fractals with lower contributions of fine patterns (see, for example, Supplementary Figure S1 where the fine scale branches are longer for the higher dimension patterns). Below we present the results of two methods used to determine the *D*_*B*_ values of each dendrite.

### 3.1 Coastline fractal analysis

**FIGURE 3:**
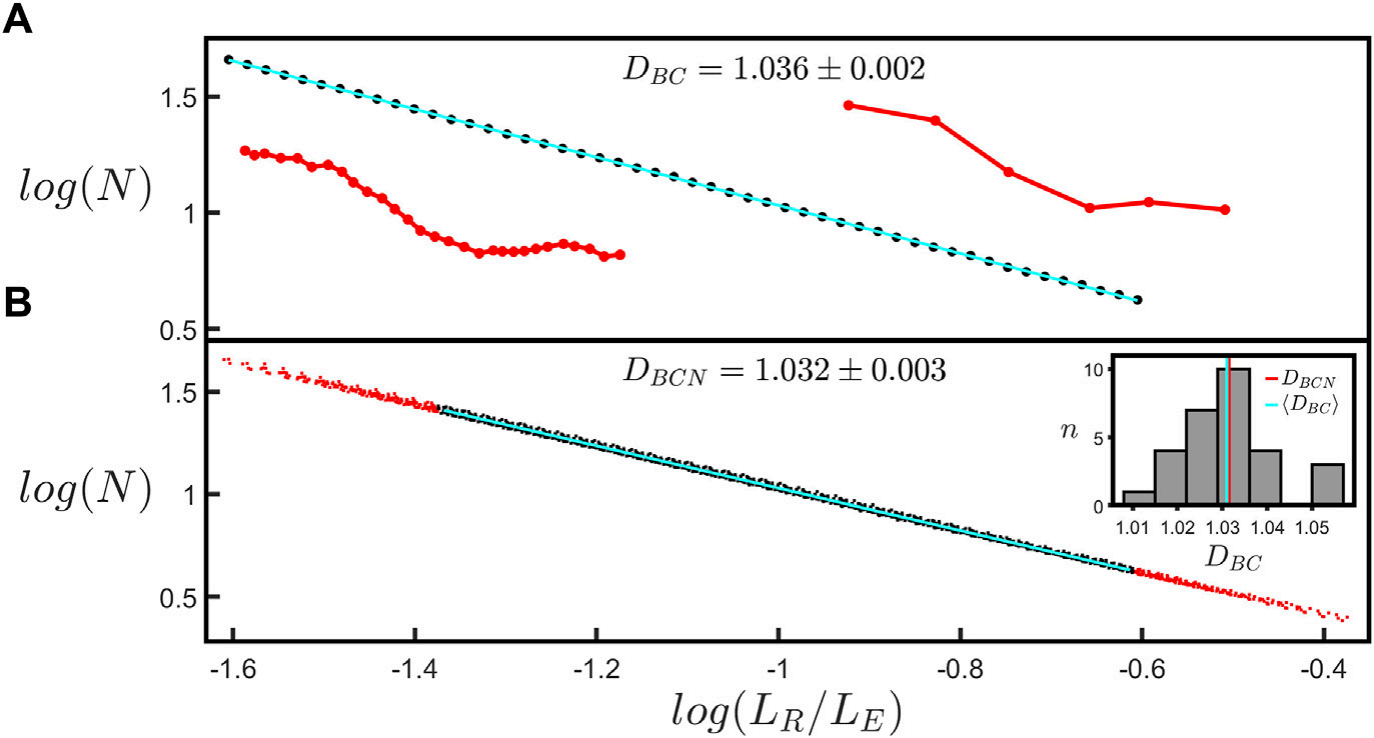
**(A)** The coastline scaling plot (base-10) of the number of rulers spanning the branch, *N*, versus the normalized ruler length, *L*_*R*_ */L*_E_, measured for a single branch within a natural neuron’s arbor. The red insets show examples of segmented versions of the branch corresponding to ruler lengths of 6.4 µm (left) and 34.7 µm (right). The slope of the line yields a coastline fractal dimension, *D*_*BC*_, of 1.036 ± 0.002. **(B)** The equivalent coastline scaling plot (base-10) including all the branches within the selected neuron’s arbor. The black data correspond to the scaling range of *L*_*R*_ */L*_*E*_ shared by all branches within the arbor, whereas the red data correspond to the range in which some branches do not contribute and are accordingly removed when fitting the data. The slope of the line yields a normalized coastline fractal dimension, *D*_*BCN*_, of 1.032 ± 0.003. The inset at the right shows a histogram of the number of branches, *n*, of a given *D*_*BC*_ within the neuron’s arbor. The vertical red and cyan lines correspond to *D*_*BCN*_ and the mean coastline fractal dimension across all the branches within the neuron’s arbor, ⟨*D*_*BC*_⟩, respectively.

The log-log (base-10) scaling plot for the “coastline” fractal analysis is shown in Figure 3A. Normalizing *L*_*R*_ using *L*_*E*_ allows scaling plots for branches with different lengths to be plotted on a common *x*-axis; Figure 3B demonstrates that all of the branches within a given arbor condense onto a single line, indicating that they are quantified by a common fractal dimension, *D*_*BCN*_. To extract *D*_*BCN*_, we focus the fit on the black data corresponding to the scaling range of *L*_*R*_ */L*_*E*_ shared by all branches within the arbor (i.e. the region over which all of the individual plots overlap). The red dots in Figure 3B indicate the data that are excluded from the fit. The inset employs a histogram to compare the mean *D*_*BC*_ across all branches within the arbor, ⟨*D*_*BC*_⟩, (1.031 as indicated by the cyan line) with *D*_*BCN*_ (1.032 as indicated by the red line). The scaling range of the fit in Figure 3A is restricted to 1 order of magnitude to provide a standardized fitting procedure for extracting *D*_*BC*_ (see Materials and methods Section 2.3). Supplementary Figure S3 shows the fit used in Figure 3A when it is extended to larger scales so that it spans 1.5 orders. The inset provides a histogram showing a comparison of *D*_*BC*_ values for fits over 1 and 1.5 orders of all branches across all neurons long enough to have a scaling range up to 1.5 orders. We will return to the limits of the scaling range in the Discussion.

**FIGURE 4:**
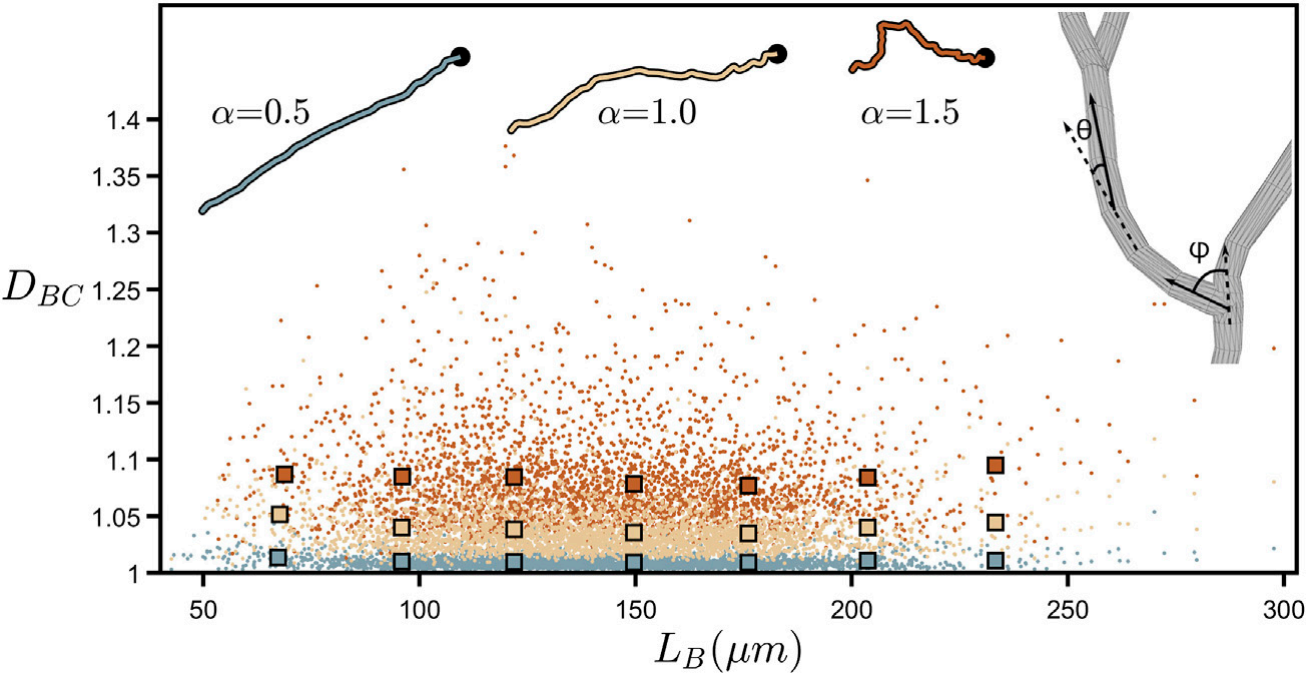
Coastline fractal dimension, *D*_*BC*_, plotted against branch length, *L*_*B*_, (measured in µm) for all of the branches across all neurons. The larger, outlined squares indicate binned averages of the underlying data in the range of 50–250 µm. The upper-right inset shows a zoom-in of a bifurcation in a neuron’s dendrites and demonstrates how the forking angle, *ϕ*, and the weave angle, *θ*, are measured. The other insets show the path of a single neuron branch for three values of the angle multiplier, *α*, where the location of the neuron’s soma is indicated by the black dot. The colors of the data shown in this plot correspond to the *α* values shown in the insets.

Figure 4 further demonstrates *D*_*BC*_’s lack of dependence on branch length by plotting the values of all the individual branches across all of the neurons examined. *L*_*B*_ is the branch’s “path length” (symbolized by the black line in Figure 2A) and is given by the total length of all the cylindrical segments spanning the branch from soma to tip (in terms of ruler measurement, *L*_*B*_ approximates to the total length of all rulers spanning the branch when the ruler is set to equal the smallest resolution possible). Although *D*_*BC*_ can vary considerably between individual branches, we note that their collective behavior reveals an independence of *D*_*BC*_ with respect to *L*_*B*_. We also mathematically manipulate the branch weave and forking angles, labelled as *θ* and *ϕ*, respectively, by multiplying every *θ* and *ϕ* value by a common factor *α* (Figure 4-right inset shows a schematic of *θ* and *ϕ*). This changes the *D*_*BC*_ values as follows. Values of *α* higher than 1 increase the angles above their natural values and cause the neuron branches to curl up, causing *D*_*BC*_ to rise because the amount of fine structure in the branch’s shape increases. Similarly, reducing *α* causes the branches to gradually straighten out, decreasing the amount of fine structure, and causing *D*_*BC*_ to drop. The insets at the top of Figure 4 provide a visual demonstration of this curling process.

### 3.2 Comparison of coastline and tortuosity fractal analysis

Whereas the coastline method considers the entire length of the branch and examines how the branch properties change with measurement resolution, our second method considers the finest resolution and examines how the branch properties vary when investigating increasingly small sections of the branch. We will show that this second approach aligns with one of the traditional measures of tortuosity, *T*, that quantifies the extent to which the meandering branch deviates from a straight trajectory. Several different tortuosity metrics have been used in previous studies of a variety of biological structures (Hart et al., 1999; Bullitt et al., 2003; Wen et al., 2009; Ledderose et al., 2014; Lorthois et al., 2014; Barbará-Morales et al., 2020). Due to its mathematical connection to fractal measurement, here we present the results of the tortuosity analysis based on $T=L_{P}/L_{D}$ (shown in Figure 2B).

**FIGURE 5:**
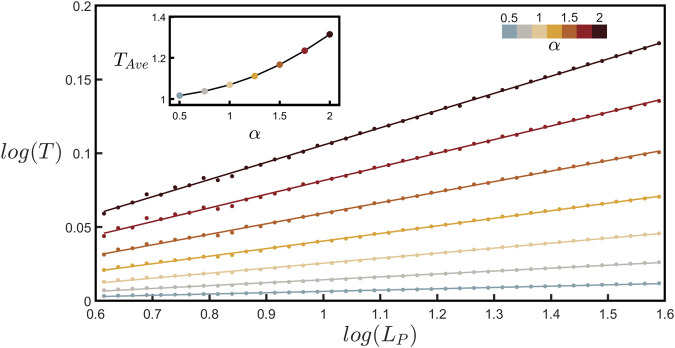
Scaling plot (base-10) of tortuosity, *T*, against path length, *L*_*P*_, (measured in µm) for seven values of *α* as indicated by the upper-right color bar. The data shown represent binned averages of *T* across all possible paths within all of the branches across all neurons. The upper-left inset shows how the average value of tortuosity across the *L*_*P*_ range examined in the main plot, *T*_*Ave*_, increases with *α*.

By measuring various path lengths, *L*_*P*_, and their corresponding displacement lengths, *L*_*D*_, along the branches of all the neurons and averaging the resulting tortuosity across all of these branches, we are able to chart the relationship between *T* and *L*_*P*_ in Figure 5 (plotted over the same scaling range used to measure *D*_*BC*_). Section 1 of the Supplementary Material derives the mathematical relationship between *T* and the coastline method and shows that for fractal behavior, *T* is expected to follow the power law relationship with *L*_*P*_ revealed in Figure 5, with the slope, *S*, of the log-log (base-10) plot related to branch fractal dimension using $D_{BT}=1/\left(1-S\right)$ . Accordingly, increasing *α* results in a steeper slope of the data line. The inset of Figure 5 confirms intuition that the *T* value averaged across the data line will increase with *α*.

**FIGURE 6:**
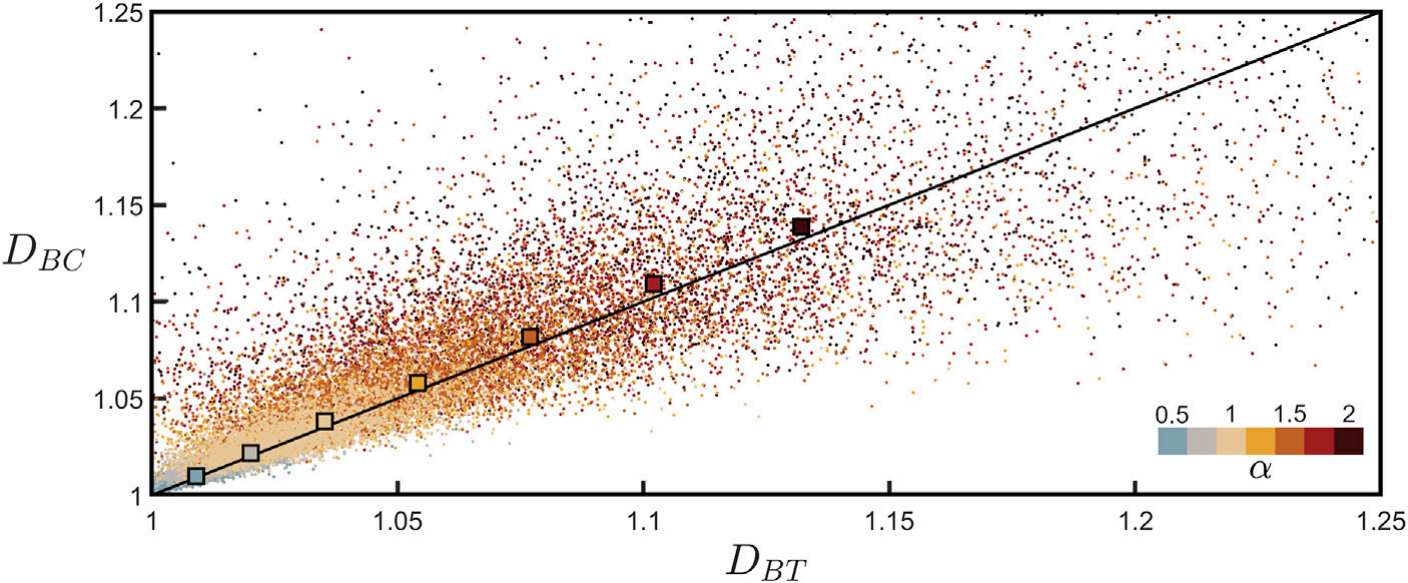
Coastline fractal dimension, *D*_*BC*_, plotted against tortuosity fractal dimension, *D*_*BT*_, for all of the branches across all neurons for seven values of *α*. The lower-right color bar indicates the *α* value of the corresponding data. The larger, outlined squares show the mean of the *D*_*BC*_ data points plotted against the *D*_*BT*_ value extracted from Figure 5 for each *α* value. The black line indicates *D*_*BC*_ = *D*_*BT*_.

Figure 6 plots *D*_*BC*_ against *D*_*BT*_ measured for all of the individual branches across all neurons and allows for a direct comparison of the two techniques used for determining branch dimension. The black line indicates the expected relationship, *D*_*BC*_ = *D*_*BT*_. To compare the data to this line, for each *α* value we also plot the *D*_*BC*_ value averaged across all branches across all neurons. Recognizing that the tortuosity scaling plots for the individual branches are inherently more noisy than the equivalent coastline scaling plots (compare Figure 3A; Supplementary Figure S2 which show the scaling behaviors for the same branch), we also plot the *D*_*BT*_ values obtained from the procedure shown in Figure 5, which benefits from fitting the combined data of all neurons. Given the scatter observed in the individual branch data points, the close match of the two techniques to the line is impressive. In addition to demonstrating the power of confirming branch dimension using two techniques, Figure 6 also emphasizes that neuron fractal behavior varies considerably from branch to branch, but nevertheless systematic behavior emerges when looking across the collective behavior of many neurons.

### 3.3 Dependence of arbor fractal dimension on branch dimension

**FIGURE 7:**
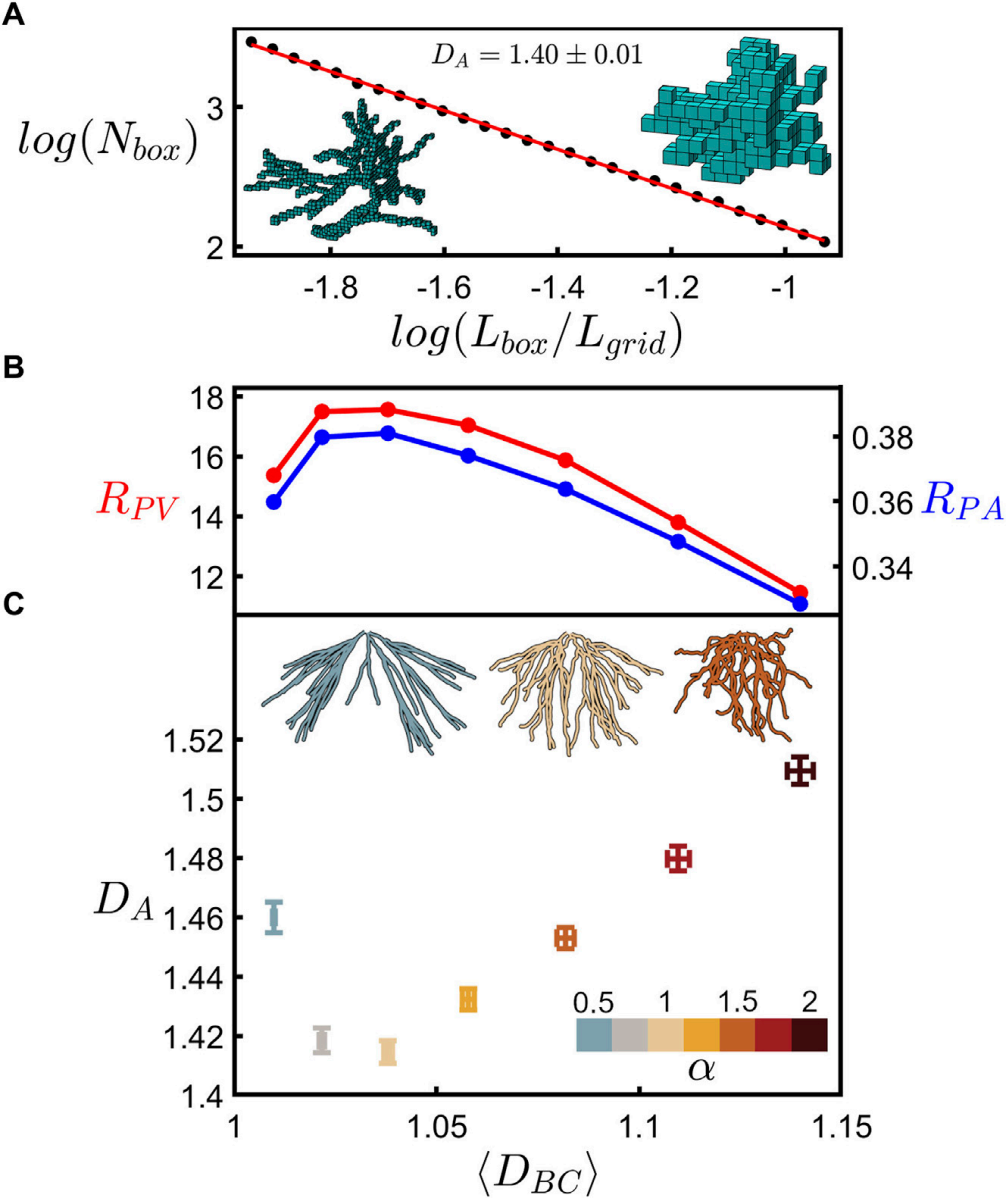
**(A)** Scaling plot (base-10) of the number of occupied boxes, *N*_*box*_, versus the normalized box size, *L*_*box*_ */L*_*grid*_, for an example neuron’s dendritic arbor. The left inset shows a neuron at a small box size (4.5 µm) whereas the right inset shows a neuron at a large box size (17.1 µm). **(B)** *R*_*PV*_ (red) and *R*_*PA*_ (blue) plotted against the mean coastline fractal dimension, ⟨*D*_*BC*_⟩. **(C)** Arbor fractal dimension, *D*_*A*_, plotted against ⟨*D*_*BC*_⟩ for seven values of *α* as indicated by the lower-right color bar. The shown data represent the mean of *D*_*A*_ and ⟨*D*_*BC*_⟩ across all arbors at each *α* value, with the error bars indicating the standard error from the mean. The three upper insets show an example neuron’s dendritic arbor for three values of *α* as indicated by the color of the arbor. **(B)** and **(C)** share the same x-axis.

Having gained certainty in our fractal branch measurements, Figure 7 shows the relationship between the fractal dimension of the neuron’s branches and the fractal dimension of its whole arbor. Details of the arbor fractal analysis are presented elsewhere (Smith et al., 2021) and so here we present a summary. Whereas the coastline method counts the number of rulers as ruler size is reduced, the analogous arbor analysis replaces the rulers with boxes to accommodate the fact that the arbors feature multiple branches. The box counting technique then determines the amount of space occupied by the arbor by inserting it into a three-dimensional grid of the boxes and counting the number of boxes, *N*_*box*_ *,* occupied by the branches. This count is then repeated across a range of box sizes, *L*_*box*_. Fractal scaling follows the power law $N_{box}\propto {L_{box}}^{-D_{A}}$ (In Figure 7A, *L*_*box*_ is normalized to *L*_*grid*_, which is the largest of the three side lengths for the smallest grid capable of enclosing the neuron being analyzed). The insets to Figure 7A show schematics of examples of small and large box sizes.

The bottom panel to Figure 7C then compares the *D*_*A*_ measurements across all the neurons to their mean branch fractal dimension, ⟨*D*_*BC*_⟩. Examining how these measurements vary with *α*, we find that both increasing and decreasing *α* results in a rise in *D*_*A*_ *.* This can be understood in terms of the interplay of fractal branches and gaps. The branches self-avoid at the natural condition of *α* = 1 and so move closer together when *α* is either increased or decreased. This is demonstrated by the insets of Figure 7C which show an example arbor for the natural case (middle) and for lower (left) and higher (right) *α* values. This generates an increase in the ratio of fine to coarse structure and a corresponding rise in *D*_*A*_.

Our previous analysis showed that *D*_*A*_ maps the balance between the neuron’s potential to connect to its neighbors and the associated operational and material costs (Smith et al., 2021). In this analysis, connectivity was assessed using an arbor’s physical profile, *P*, since large profiles result in the increased exposure of synapses (which are responsible for receiving signals from other neurons). “Operating” costs were assessed using the surface area of the dendrites comprising an arbor, *A*_*s*_, based on research of neuron ATP energy expenditures (Attwell and Laughlin, 2001; Wen et al., 2009). The volume occupied by an arbor’s branches, *V*_*m*_, was used to quantify the “building” costs of the arbor. Both *P* and *A*_*s*_ were normalized to the bounding area, *A*_*b*_, of an arbor, while *V*_*m*_ was normalized to the bounding volume, *V*_*b*_, of an arbor to ensure that our measures of connectivity, operating cost, and building cost do not depend on the overall size of the arbor.

The ratios of the rates of change of connectivity with operating cost

$$R_{PA}=\frac{\frac{d}{dD_{A}}\left(\frac{P}{A_{b}}\right)}{\frac{d}{dD_{A}}\left(\frac{A_{s}}{A_{b}}\right)}$$

and with building cost

$$R_{PV}=\frac{\frac{d}{dD_{A}}\left(\frac{P}{A_{b}}\right)}{\frac{d}{dD_{A}}\left(\frac{V_{m}}{V_{b}}\right)}$$

Then quantified how the arbors balance these factors as a function of *D*_*A*_.

Specifically, it was suggested that peaks in *R*_*PA*_ and *R*_*PV*_ indicated the optimal balance. Supplementary Figure S4 plots the *D*_*A*_ dependences of *P/A*_*b*_, *A*_*s*_ */A*_*b*_, and *V*_*m*_ */V*_*b*_ corresponding to the data in Figure 7C and shows that, as expected from the previous investigation, *R*_*PA*_ and *R*_*PV*_ both peak at around the natural neurons’ prevalent *D*_*A*_ value. Figure 7B is generated by first using Figure 7C to convert the ⟨*D*_*BC*_⟩ values for each *α* value to their associated *D*_*A*_ values and then using Supplementary Figures S4D, E to convert these *D*_*A*_ values to their *R*_*PA*_ and *R*_*PV*_ values. As shown by the falling *R*_*PA*_ and *R*_*PV*_ values, the balance between connectivity and cost deteriorates as the *D*_*A*_ value moves to the higher, unnatural values.

Figure 7 emphasizes that even for neurons composed of dendrites with very mild fractality (characterized by low dimensions close to those of Euclidean straight lines and charted over just one order of magnitude), ⟨*D*_*BC*_⟩ nevertheless serves as a key parameter for charting the interplay between the arbor branches and their gaps, resulting in a systematic shift from natural to non-optimal *D*_*A*_ values. In terms of the sensitivity of neuron behavior to changes in their ⟨*D*_*BC*_⟩ values, we draw attention to the asymmetry of the curves in Figures 7B, C. Distortions that increase the dendrites’ weaving and forking angles lead to small increases in *D*_*A*_ compared to the sharper rises observed for distortions that reduce these angles. In particular, arbors featuring dendrites close to the Euclidean condition are highly sensitive to distortions. For example, the small reduction in ⟨*D*_*BC*_⟩ from 1.02 (*α* = 0.75) to 1.01 (*α* = 0.5) is accompanied by an increase in *D*_*A*_ from 1.42 (*α* = 0.75) to 1.46 (*α* = 0.5)—relative to the dendrites, the arbor’s dimension increases approximately fourfold. The associated reductions in *R*_*PA*_ and *R*_*PV*_ values exhibit similar sensitivities to *D*_*BC*_.

## 4 Discussion

Fractal geometry is a useful model for capturing the multi-scaled complexity of many natural objects and distinguishes their resulting pattern characteristics from single-scaled “Euclidean” objects. In this paper, we have refined our fractal investigations of neurons by further clarifying the origin and extent of their fractal properties. The arbor’s underlying scaling properties arise from variations in the weave, forking, and length distributions of its branches and this structural interplay results in a more intricate scaling behavior than that exhibited by simple fractal models (for example, the H-Tree shown in Supplementary Figure 1). Because fractal dimension, *D*, is sensitive to how all three branch parameters shape the neuron’s underlying geometry, it can be related to more traditional parameters used to study specific consequences of the neuron complexity.

This was highlighted by our comparisons between *D* and tortuosity, *T*. Defining *T* as the ratio of path length to the Euclidean distance between the path’s start and end points, *T* can be used to quantify the weave of an individual branch measured at a specific size scale. In contrast, *D* captures a more comprehensive picture by accounting for the weave’s power law growth in tortuosity across increasingly large scales. This was demonstrated here by deriving the mathematical relationship between branch tortuosity and a traditional measure of fractal scaling, and then using our neuron models to confirm the agreement between the two associated dimensions, *D*_*BT*_ and *D*_*BC*_. We note that one consequence of our definition of a branch (any path that starts from the soma and ends at the tip of a dendrite) is that different branches commonly have shared sections (Figure 1E). As such, each of these shared sections influences the calculation of *D*_*BC*_ for multiple branches. This is appropriate because an entire neuron’s geometry will inherently be more dependent upon these shared sections (for example, a section close to the soma might influence the location of many branch tips).

To clarify the origin of the neuron’s fractal behavior, we examined how the individual branches relate to the fractal properties of the whole arbor. To do so, we considered two distinct fractal dimensions - *D*_*B*_ and *D*_*A*_ which quantify the scaling properties of a branch and of an arbor, respectively. Our results show that the branches in an arbor typically exhibit the same fractal behavior irrespective of their length within the measurable fractal range. Furthermore, within scatter, branches from different neurons exhibit this common behavior. Surprisingly, they all reveal very mild fractal characteristics quantified by low *D*_*B*_ values close to those expected for straight lines. As these branches spread out in space, the resulting arbor properties depend on two embedded fractal patterns—the branches and the gaps forming between them (as highlighted by the insets in Figure 7C). This spatial relationship generates a much larger complexity than that of the individual branches. We anticipate that the resulting U-shaped relationship between *D*_*B*_ and *D*_*A*_ will be generic to neuron types with arbors composed of fractal branches that self-avoid. For such neurons, this fractal structure optimizes the balance between connectivity and operating costs, as indicated by the deterioration in the *R*_*PA*_ and *R*_*PV*_ values shown in Figure 7B. For neurons with a spread of *D*_*A*_ values, we expect an analogous behavior to that shown in Supplementary Figure 3 whereby *R*_*PA*_ and *R*_*PV*_ peak at *D*_*A*_ values closely matching the histogram peak, indicating that the majority of neurons exist near the optimizing condition.

In terms of distortions away from the natural fractal condition, it is informative to examine the symmetry of the U-shape. For the hippocampal neurons of the current study, the natural condition (*α* = 1) centered around *D*_*B*_ = 1.04 and distortions that reduced the fractal weave of the branches resulted in relatively large changes to the arbor fractal characteristics as the branches neared the Euclidean condition of straight lines. Based on this observation, we anticipate that neuron types with naturally occurring low *D*_*B*_ values that are distorted in a manner that reduces their weave or forking angles will experience large changes in their arbor fractal characteristics and associated functionality. This however assumes a similar arbor density to the neurons examined in the current study - this behavior may not be seen for sparsely branching neurons. It is also intriguing to consider neurons with large naturally occurring *D*_*B*_ values and examine whether distortions through increases in their weave and forking angles would experience a similar sensitivity to *D*_*B*_ *.* We hope that the current study will encourage analogous future studies across different neuron types that investigate these behaviors.

In addition to facilitating connections between research from different fields (in particular, biology and mathematics), a powerful motivating factor for using two measurement techniques (the coastline and tortuosity techniques) is to provide confirmation that the branches are indeed described by a fractal dimension. This is an important capability in light of the importance of distinguishing the branches fractal behavior from the Euclidean behavior characterized by integer dimensionality. Because the observed *D*_*B*_ values are so close to one-dimensional behavior and are observed over a highly limited scaling range, it is valuable to consider our results within the context of a larger debate within fractal studies—when is it appropriate to label behavior as fractal? In declaring “The Fractal Geometry of Nature” (Mandelbrot and Pignoni, 1983), Mandelbrot introduced fractality as an umbrella terminology to unite studies of scale-invariant behavior in physical and mathematical systems. He did not introduce scaling range into the definition of fractality in part because of the contrast between the infinite repetition of mathematical patterns and the limited repetition of physical patterns, but crucially because the scaling range necessary for fractality to impact functionality varies considerably between physical objects.

A subsequent survey revealed that published experimental studies of fractality in physical systems typically displayed scale-invariance over only 1.3 orders of magnitude (Avnir et al., 1998). Guidelines from the survey authors for whether scaling plots with limited range are useful included: 1) “[it] condenses the description of a complex geometry”, 2) “It allows one to correlate in a simple way properties and performances of a system to its structure.” Returning to our current studies, neuron branches are inevitably limited in their scaling capacity due to the finite branch segment sizes at the fine scale and the finite arbor sizes at the course scale: based on the 4 µm fine scale cut-off and the mean branch length of 141 μm, the capacity for fractality is limited to approximately 1.5 orders of magnitude. Nevertheless, Figure 7 satisfies the above two criteria for usefulness by displaying a clear link between ⟨*D*_*BC*_⟩ and neuron functionality. Furthermore, despite being close to the one dimensionality of straight lines, the fractional dimension values measured for the mildly weaving branches translate to different *R*_*PV*_ and *R*_*PA*_ values than for the Euclidean condition and therefore signal different functionality. Whilst acknowledging the potential for controversy, the limited range and low dimensions of the neurons could be viewed as a demonstration that even mild fractality can have an important impact on functionality. That said, future studies should compare our fractal approach to other biomarkers to further explore its usefulness.

It is interesting to view the arbor’s fractal complexity in terms of an emergent property of the system (Coveney and Highfield, 1995) when small changes in the components (charted by *D*_*B*_) have the potential to generate large changes in the interactions of the system (charted by *D*_*A*_). In addition to being of interest to fundamental neuroscience research, this sensitivity should be taken into account when developing applications. Here, we briefly consider two applications that focus on deviations from natural neuron behavior. Based on the *D*_*A*_ vs. ⟨*D*_*BC*_⟩ dependence of Figure 7C, pathological conditions that modify the branch weave have the potential to induce radical changes in arbor structure and its connectivity within a neural network; Figure 7 suggests that the impact of the changes will depend on the neurons’ naturally occurring ⟨*D*_*BC*_⟩ value and the specific manner in which the branch fractal character changes. Many neurological conditions are associated with neuron pathology, and a fractal method capable of identifying specific changes in the dendritic arbor may be a useful tool in early detection of the prodromal stages of these diseases. For example, the neurodegenerative process in Alzheimer’s disease includes atrophy of the dendritic arbor of neurons in many parts of the limbic system and cortex (Serrano-Pozo et al., 2011). However, studies often focus on changes in dendritic spines only, whereas changes in neuron morphology are reported only as a general reduction of branching complexity (Cochran et al., 2014). Analysis of the fractal characteristics of these pathological neurons may provide biomarkers for their early detection, similar to how fractal dimension can differentiate the stages of cancer (Elkington et al., 2022). It is important to note that high resolution imaging would likely be required to accurately detect prodromal changes affecting a neuron’s fractal characteristics, as well as automation to characterize large numbers of samples (Rowland et al., 2022). As such, a goal of future studies is to apply our technique to publicly accessible repositories of images from experiments, for example online libraries such as NeuroMorpho.Org (Ascoli et al., 2007) with a much larger number of neurons featuring a range of pathological conditions. Given that our mathematical models benefit from generating distortions in the fractal weave whilst holding other variables constant, the current investigation can be seen as the initial controlled demonstration ahead of these experimental challenges.

There is also growing interest in quantifying neuron interactions with artificial surfaces, for example for incorporation into implants in the human body. In particular, advances in device fabrication capabilities allow surfaces to be physically patterned to direct the growth of the neurons as they connect to form a network on the surfaces (Jang et al., 2010; Piret et al., 2013; Diaz Lantada et al., 2015; Yiannakou et al., 2017; Moslehi et al., 2020; Moslehi et al., 2022). This includes changing the direction of the neuron branches, such as directing them along straight lines. These directional changes can be viewed as the physical analogy of our distortions of the weave and forking angles. As such, even small changes in the branch weave might shift the network away from an optimal balance of connectivity versus cost. The results of Figure 7 suggest that implants should match their surface patterning to that of the neurons’ fractal geometry to maintain the natural performance of the network. This “resonance” between the artificial and natural fractals can be seen as a novel form of biocompatibility and adds geometry to the traditional research of materials and chemical environments.

## Data Availability

The data presented in the study are deposited in a GitHub repository at https://github.com/conor-rowland/hippCA1neuronreconstructions.
