# Supplementary material for: Neuron arbor geometry is sensitive to the limited-range fractal properties of their dendrites
Source: Front Netw Physiol. 2023 Jan 25;3:1072815. doi: 10.3389/fnetp.2023.1072815 (PMC10013056; doi:10.3389/fnetp.2023.1072815)
Supplement: Supplementary file 1 [file DataSheet1.docx]

Supplementary Material

# Derivation of Tortuosity Fractal Dimension, *D_BT_*

The fractal dimension, *D_B_*, of a selected branch section can be quantified using the power law dependence of the number of rulers, *N*, spanning the branch section and the ruler length, *L_R_*, being used:

|  | $N\propto\left( \frac{L_{R}}{L_{D}} \right)^{-D_{B}}$, | (Eq. 1) |
| --- | --- | --- |

where *L_R_* has been normalized to the ruler length spanning the chosen section *L_D_*.

For the traditional coastline method (for which we label *D_B_* as *D_BC_*), the full branch length is considered (i.e. *L_D_* = *L_E_*) and the ruler count is measured as *L_R_* is reduced, generating the log-log plot of Figure 3A with a slope of -*D_BC_*.

For the tortuosity method (for which we label *D_B_* as *D_BT_*), count variations are converted to length variations by substituting *N* = *L_T_*/*L_R_* into Eq (1):

|  | $L_{T}\propto{L_{R}}^{-\left( D_{BT}-1 \right)}{L_{D}}^{D_{BT}}$ , | (Eq. 2) |
| --- | --- | --- |

Whereas the coastline method sets the second term of Eq. 2 constant and investigates the dependence on *L_R_*, the tortuosity method sets the first term constant by considering a ruler length set at the finest resolution (i.e. *L_R_* = *L_F_* and therefore *L_T_* = *L_P_*) and investigates the changes in *L_P_* as *L_D_* is reduced. This generates the following relationship:

|  | $L_{P}\propto C{L_{D}}^{D_{BT}}$ , | (Eq. 3) |
| --- | --- | --- |

where the constant, *C*, is given by

|  | $C={L_{F}}^{-\left( D_{BT}-1 \right)}$ . |  |
| --- | --- | --- |

Rearranging Eq. 3 and substituting in the definition of tortuosity *T* = *L_P_/ L_D_* then gives

|  | $T\propto{L_{P}}^{\frac{D_{BT}-1}{D_{BT}}}$ . | (Eq. 4) |
| --- | --- | --- |

Labelling the slope of the log-log plot of *T* vs *L_P_* as *S* then:

|  | $D_{BT}=\frac{1}{1-S}$ . | (Eq. 5) |
| --- | --- | --- |

# Supplementary Figures and Tables


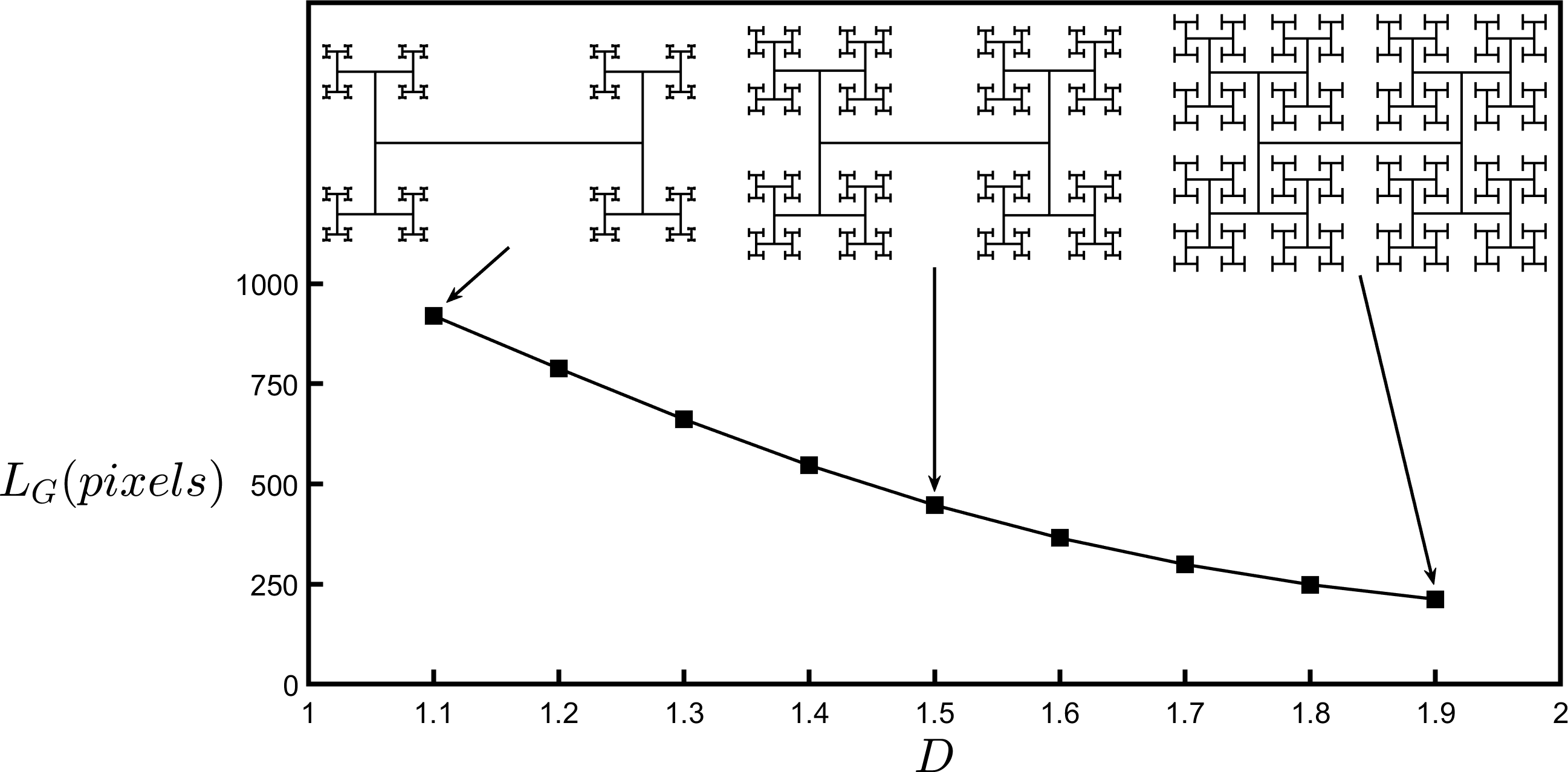


**Supplementary Figure 1.**  Demonstration of the impact of the fractal dimension of H-Tree patterns on the gaps between their branches. Plot of *L_G_* (the distance between each gap pixel and the nearest branch pixel averaged across all gap pixels where gap pixels are limited to those within the convex hull of the H-Tree) against *D*. The insets show three H-Tree patterns with *D* = 1.1 (left), 1.5 (middle) and 2 (right).


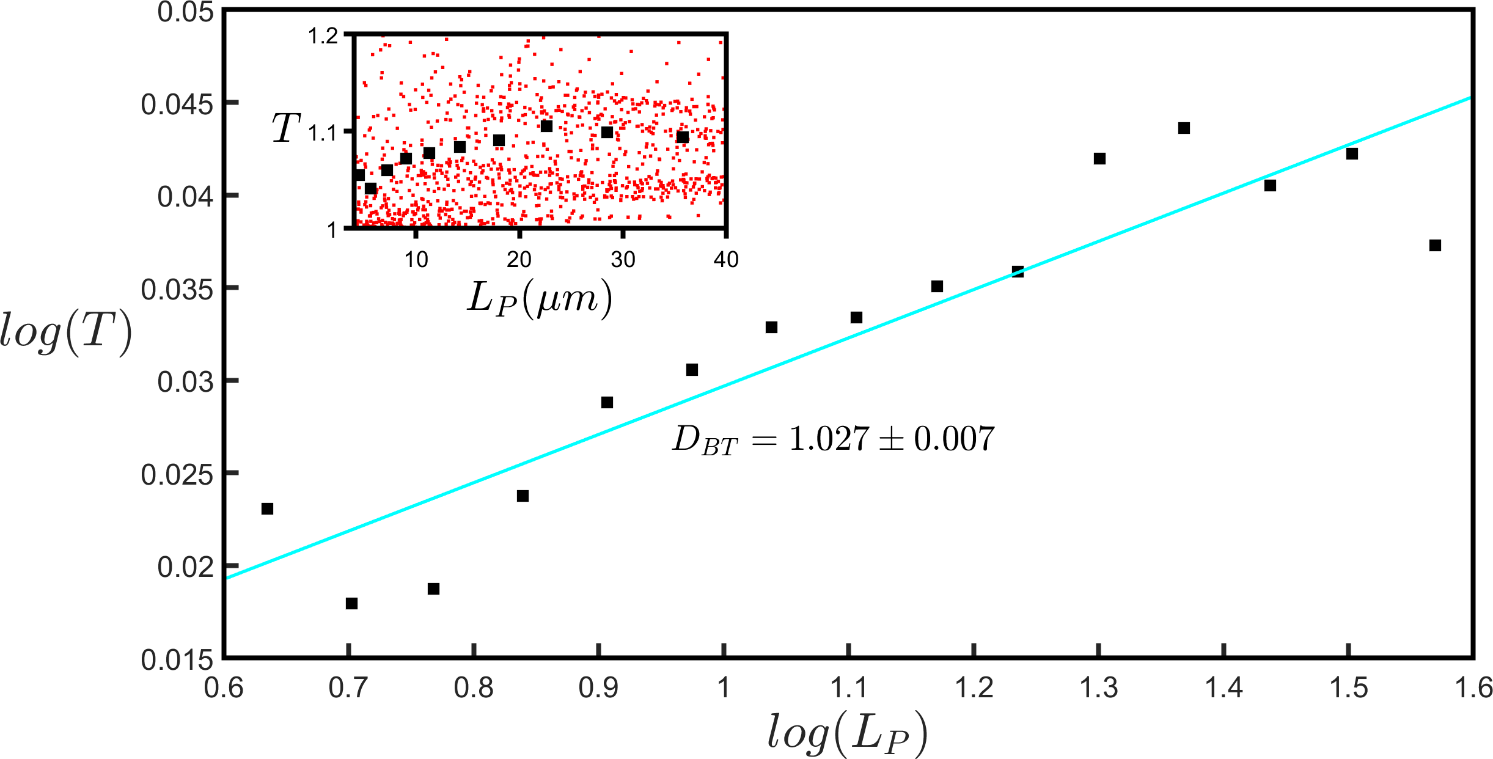


**Supplementary Figure 2.** The scaling plot (base-10) of tortuosity, *T*, versus path length, *L_P_*, (measured in µm) for the same branch shown in Figure 3A. Each black square represents the average *T* value of all possible paths within the branch for the given *L_P_* value. The cyan line shows the fit used to calculate *D_BT_*. The inset demonstrates the averaging procedure by plotting *T* versus *L_P_*, where each red dot represents a single path and the black squares represent their binned averages (which correspond to those shown in the main plot).

**
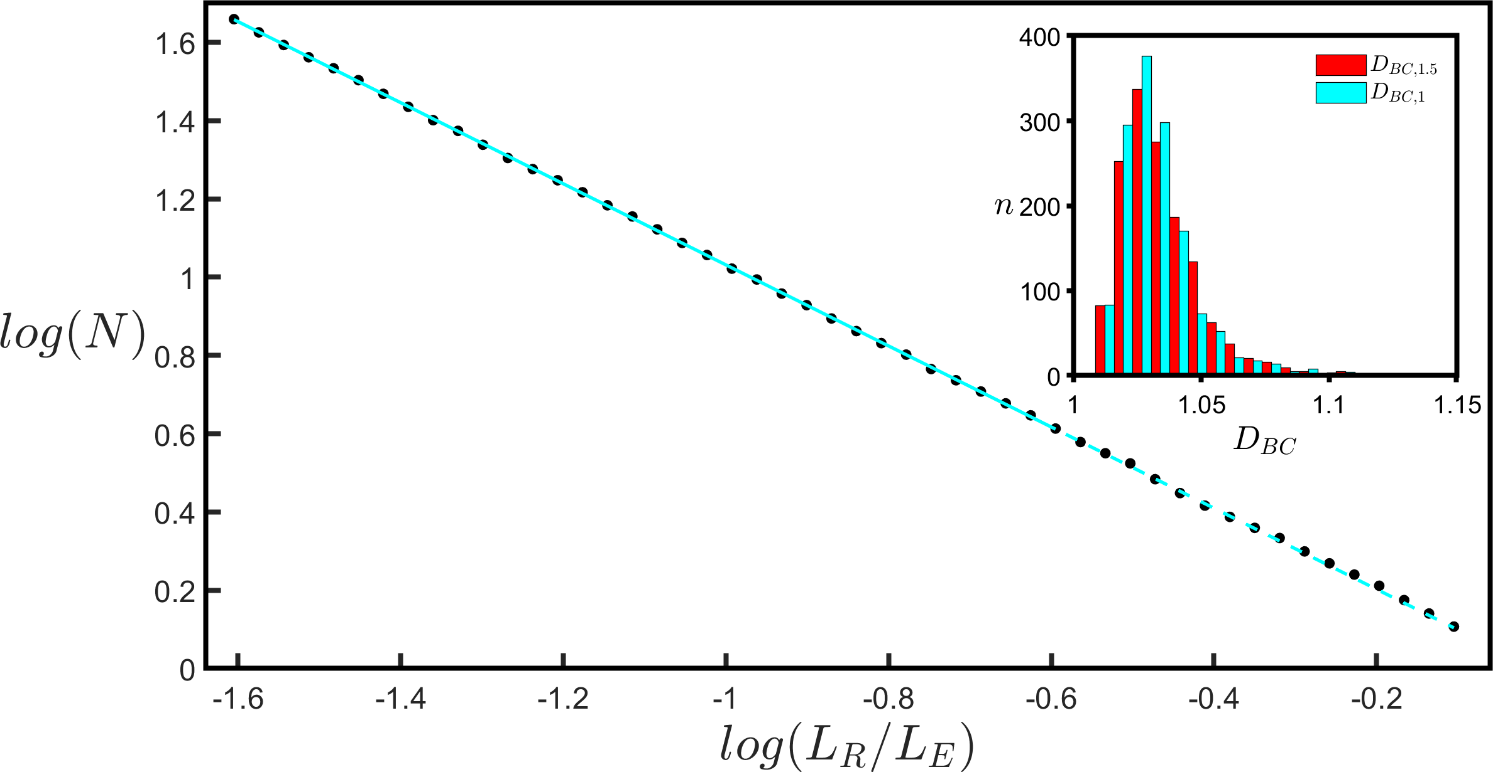
**

**Supplementary Figure 3.** The coastline scaling plot (base-10) of the number of rulers spanning the branch, *N*, versus the normalized ruler length, *L_R_*/*L_E_*, measured for the same neuron branch shown in Figure 3A. The solid cyan line represents the fit used to extract *D_BC_* over the 1 order of magnitude scaling range seen in Figure 3A, while the dashed cyan line represents the extension of this fit up to 1.5 orders of magnitude. The upper-right inset shows a histogram comparing the *D_BC_* values extracted from fits over 1 (cyan) and 1.5 (red) orders of magnitude for all branches across all neurons long enough to have a scaling range up to 1.5 orders.


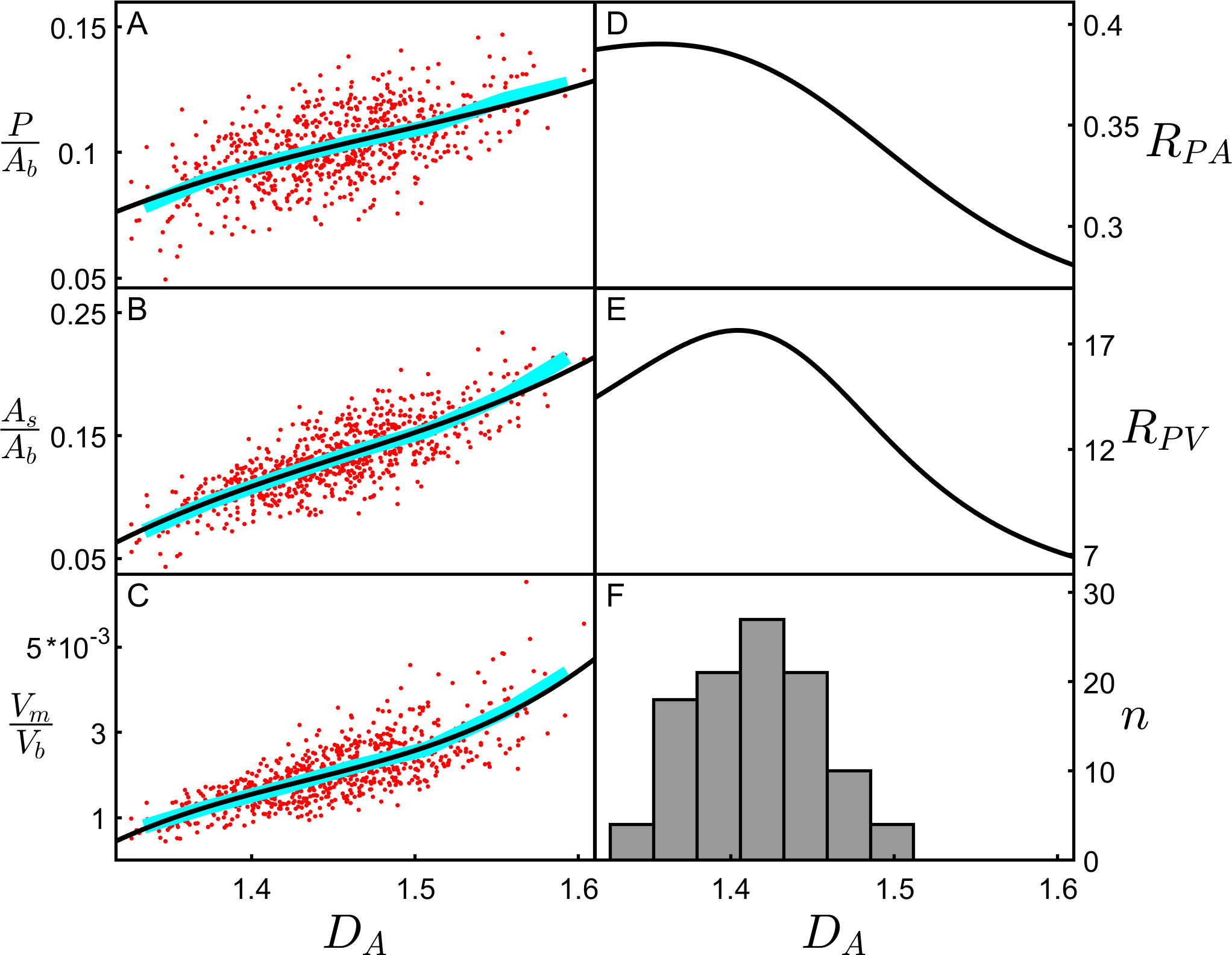


**Supplementary Figure 4.** Left column. Dependences of *P/A_B_* (**A**), *A_s_/A_b_* (**B**) and *V_m_/V_b_* (**C**) on *D_A_*. The underlying red data includes both natural and distorted neuron arbors, the cyan lines correspond to binned averages of the data, and the black curves correspond to 3^rd^ degree polynomial fits to the data. Right Column. (**D**) *R_PA_* (the ratio of the derivatives of the fits to *P/A_b_* and *A_s_/A_b_*) plotted against *D_A_*. (**E**) *R_PV_* (the ratio of the derivatives of the fits to *P/A_b_* and *V_m_/V­­_b_*) plotted against *D_A_*. (**F**) Histogram of *n*, the number of natural neurons with a given *D_A_* value.
